# Asking questions that matter – Question prompt lists as tools for improving the consent process for neurotechnology clinical trials

**DOI:** 10.3389/fnhum.2022.983226

**Published:** 2022-07-29

**Authors:** Andreas Schönau, Sara Goering, Erika Versalovic, Natalia Montes, Tim Brown, Ishan Dasgupta, Eran Klein

**Affiliations:** ^1^Department of Philosophy, University of Washington, Seattle, WA, United States; ^2^Dana Foundation, New York, NY, United States; ^3^Department of Neurology, Oregon Health & Science University, Portland, OR, United States

**Keywords:** BCI (brain computer interface), DBS (deep brain stimulation), QPL (question prompt list), neurotechnology, participant perspective

## Abstract

Implantable neurotechnology devices such as Brain Computer Interfaces (BCIs) and Deep Brain Stimulators (DBS) are an increasing part of treating or exploring potential treatments for neurological and psychiatric disorders. While only a few devices are approved, many promising prospects for future devices are under investigation. The decision to participate in a clinical trial can be challenging, given a variety of risks to be taken into consideration. During the consent process, prospective participants might lack the language to consider those risks, feel unprepared, or simply not know what questions to ask. One tool to help empower participants to play a more active role during the consent process is a Question Prompt List (QPL). QPLs are communication tools that can prompt participants and patients to articulate potential concerns. They offer a structured list of disease, treatment, or research intervention-specific questions that research participants can use as support for question asking. While QPLs have been studied as tools for improving the consent process during cancer treatment, in this paper, we suggest they would be helpful in neurotechnology research, and offer an example of a QPL as a template for an informed consent tool in neurotechnology device trials.

## Introduction

Implantable neurotechnology devices such as Brain Computer Interfaces (BCIs) and Deep Brain Stimulators (DBS) are an increasing part of treating or exploring potential treatments for neurological and psychiatric disorders. While only a few devices are approved, many promising prospects for future devices are under investigation. The decision to participate in a clinical trial can be challenging. Among other things, potential participants face a variety of challenges, including surgical risks (Fenoy and Simpson, 2014) and uncertainty about post-trial care (Hendriks et al., 2019; Sankary et al., 2021).

Over the last 10 years, our group gained experience working with people with amyotrophic lateral sclerosis (ALS) (Versalovic et al., 2020; Versalovic and Klein, 2020), Parkinson’s Disease (Wexler et al., 2022), essential tremor (Brown et al., 2016), and obsessive compulsive disorder (OCD)/depression (Klein et al., 2016). During this time, we have found that neurotechnology device trials raise a number of issues for informed consent that are not traditionally included in existing informed consent discussions or are not fully appreciated in the process. These include issues such as agential changes in the ethical dimensions of privacy, authenticity, responsibility, trust (Schönau et al., 2021), as well as relational effects (Goering et al., 2017). Quotes from two participants from our prior work illustrate the difficulty of understanding those and others challenges before entering the trial:

Participant A “I think I understood as much as I was going to understand without being a part of it. I tried to ask all the questions that I could, but I don’t think anything prepares you… I don’t think you can ask the right questions without being a part of it. I learned the science whilst I was a part of it.”

Participant B “I probably would have wanted to talk to somebody that was already involved to see what their experience was like. But as it turned out, there’s not that many people doing this.”

When it comes to communicating the challenges of a neurotechnology study during the informed consent process, it is important to recognize that potential participants might not consider – or have the language to consider – potential issues prior to enrolling in a clinical trial. At the same time, researchers themselves may struggle to identify and talk about them. Participants’ expectations of a research trial during the consent process might be very different from the actual experience they have while being in the research trial. In hindsight, they might feel unprepared or wish that certain insights of what it feels like to use a device would have been shared with them before enrollment. At the same time, there are only a few participants in studies that are usually small and spread across institutions, which makes it hard for them to share their experience with others. There is a risk that topics that might be important to future participants’ informed decision-making are not being addressed. This is why it is crucial to have practices in place that allow potential participants to engage in a more active role during the consent process for enrolling in a clinical trial.

One tool to help empower participants to play this more active role is a Question Prompt List (QPL). QPLs are communication tools that help participants and patients to articulate difficult concerns within the informed consent process (Dimoska et al., 2008). They offer a structured list of disease, treatment, or research intervention-specific questions that patients or prospective research participants can use as prompts for question-asking during the informed consent process. While QPLs have been used and tested in cancer treatment and clinical trials, their potential benefit for patients and research participants using novel neural devices has not been explored.

In this brief perspective paper, we present QPLs as a promising informed consent tool in clinical trials for neural devices. In see section “current research on question prompt lists,” we review recent research on QPLs. See section “the potential of question prompt lists in neurotechnology research” explores the potential of QPLs as an informed consent tool for non-standard issues in neurotechnological studies, specifically BCI and DBS studies. In see section “example of question prompt list tool for implanted neurotechnology studies,” we offer an example of a QPL that could serve as a template for an informed consent tool in neurotechnology device trials.

## Current research on question prompt lists

The process of making a decision for or against treatment of a disease or whether to enroll in a clinical study is a difficult one. The lack of clinical knowledge, individual hopes and fears, as well as the uncertain nature of the whole endeavor can make the process of coming to a decision burdensome. Before patients enroll in clinical studies, they engage with researchers (who may also be their clinicians) who explain and answer questions about the trial that they might enter. However, potential participants may not know what to ask or might lack the language for expressing emerging concerns.

Question prompt lists offer research intervention specific questions that can help participants to identify and talk about those concerns during the informed consent process. Over the last two decades, QPLs have primarily been studied as tools for improving the consent process during cancer treatment. QPLs have been found to increase the total number of questions asked (Butow et al., 1994; Brown et al., 1999, 2001; Clayton et al., 2003, 2007). Most patients describe QPLs as helpful and useful to help them ask more questions (McJannett et al., 2003; Langbecker et al., 2012), value them to gather new trial information (Brown et al., 2011a), and generally endorse their early implementation into the consent process of active cancer treatment (Sato et al., 2021). In relation to cancer research, QPLs have been shown to increase treatment-related knowledge and reduce patient’s decisional conflict (Jayasekera et al., 2020). They allow the patient to play a more active role during the consent process through creating an environment conducive to shared decision making (Hoffmann et al., 2014).

Such studies strongly indicate that QPLs hold promise for empowering participants in the consent process. Due to their capacity to model what can be asked, QPLs may allow participants to have a better understanding of what it is like to be treated, develop a set of reasonable expectations of their future role, and allow them to play an active role during the consent process.

## The potential of question prompt lists in neurotechnology research

One field that is particularly promising for the employment of QPLs as a tool for clinical trials is the field of neurotechnology research. Agreeing to take on an implantable neural device (such as a BCI or DBS) is momentous, given the significance of the brain for our sense of self, identity and agency (Schönau et al., 2021). In addition, making an informed decision to participate in a clinical trial with an implantable neural device can be challenging due to the range of other considerations involved, such as surgical risks (Fenoy and Simpson, 2014), or uncertainties about post-trial care (Hendriks et al., 2019). Recently, there is increased academic awareness about the need for better informed consent to get at issues of exit from a research study (Lázaro-Muñoz et al., 2018; Sankary et al., 2021). While details about those issues might already be in current informed consent documents, they might not be well understood or appreciated by potential participants. Encouraging them to play a more active role by asking questions could make those discussions easier. QPLs could help to prompt prospective participants about issues in the informed consent document that were unclear or are in need of clarification.

Beyond those concerns that are mostly addressed during the informed consent process but might need a better approach to be fully understood, neurotechnology studies provide another layer of potential issues that are not traditionally included in standard informed consent discussions. Among others, worries that go beyond that scope involve several dimensions of agency that can be impacted when participants are using a neurotechnological device. In the neuroethical literature, agential disruptions end users might experience are discussed as issues of responsibility, privacy, authenticity, trust (Schönau et al., 2021) and as relational effects (Klein et al., 2016). Responsibility is discussed under the framing of the responsibility gap, i.e., the unclarity of who is responsible for the unintended outcome of a BCI mediated movement (see, among others Grübler, 2011; Kellmeyer et al., 2016; Steinert et al., 2018; Schönau, 2021). Privacy is discussed as protecting brain data from unwanted access and establishing and negotiating boundaries (see, among others, Allen, 2014; Pyrrho et al., 2022). Authenticity is discussed as the risk of unintended changes of the self through neurostimulation (see, among others, Schüpbach et al., 2006; Kraemer, 2013; Pugh et al., 2017; Gilbert and Viaña, 2018). Trust denotes the difficulty to gain ownership or a sense of embodiment over a neurotechnological device (see, among others, Heersmink, 2011; Collins et al., 2017; Tbalvandany et al., 2019). Those non-standard issues of neurotechnology trials are wide ranging and might vary widely across studies.

People who are considering enrolling in a research trial with a BCI or a DBS might be unaware of the debate over those ethical dimensions and the agential changes they might experience after the device is implanted. And yet, during the deliberation phase for or against trial participation, they are confronted with the difficult task of imagining what it is like to have a device implanted in their head and what it feels like to actually live and act with it. Due to the novelty of the study and the limitations regarding relevant personal experience, they might lack not only the knowledge but also the language for asking about issues that are related to potential changes in their agency before enrolling in the trial.

This knowledge gap could be diminished by offering a QPL as a tool for the potential participant to ask questions informed by changes and experiences others have reported before them. While a QPL is not on par with the experience of what it is like to participate in the actual study, it can help prospective participants to know what to ask when entering the study. A well implemented QPL has the potential to facilitate enrollment of better informed participants who are more motivated throughout the trial, feel confident about what lies ahead of them, and have a better understanding about what happens when the trial ends. As such, QPLs can function as tools that help participants to imagine themselves during those trials in a more robust way.

## Example of question prompt list tool for implanted neurotechnology studies

In order to improve the consent process for people who consider enrolling in a BCI or DBS trial, we advocate for creating a QPL that encourages them to ask clarifying questions about risks introduced in the informed consent form as well as about non-standard ethical issues and experiences others might have reported while being in a similar clinical trial. As a starting point for discussion, we developed a preliminary QPL that can be used during the informed consent process of BCI/DBS trials. We directly modeled our pilot based on a QPL by Brown et al. (2011b) that has been used to improve the decision making process for enrolling in cancer clinical trials. We then modified that QPL based on qualitative interviews we conducted over the last 10 years, attending to different kinds of experiences participants report having.

This set of questions is not intended to be settled or comprehensive, but should be taken as illustrative of the kind of tool researchers could develop for neural device trials. We kept the QPL general because relevant issues to consider might vary widely across studies. From this initial model, we aim to gather feedback from and encourage discussion with participants, researchers, and stakeholders to continue revising and refining this QPL template.

### Question prompt list for person considering sensorimotor brain computer interface study participation

#### Understanding the study’s purpose and background

1. What is the purpose of this study?

2. What is already known about the technology/device being used in this study?

3. How experienced are you and your team with this device? With running this kind of study?

#### Understanding the alternatives

4. What makes me eligible (or not) for this study?

5. Are there other studies that I am eligible for?

6. If I participate in this study, will I not be eligible for studies involving future (next generation) BCIs?

7. Is access to the device only available through joining the study?

#### Understanding the possible benefits

8. What benefits could I possibly get if I join the study?

9. If I join this study, how might others benefit?

10. Have others like me benefited from participating in similar studies? If so, how?

#### Understanding the possible risks and burdens

11. Are there any long-term or permanent side effects from the surgery or from using the technology/device?

12. Are there any serious or rare side effects that I should know about?

13. Who can I call if something goes wrong?

14. If I get a side effect or injury because of being in the study, will I get compensation?

15. Will I have control over who has access to brain data collected by the device?

#### Learning from the experiences of others

16. How do participants of this and similar studies describe the experience of using the device?

17. Have other participants described what it feels like to control or struggle to control devices using the BCI?

18. Have participants in such studies talked about feeling unlike themselves, or somehow less authentic? Has this affected how individuals view/understand themselves or are viewed by others?

19. Have other participants described how the study has affected their family members?

20. Have participants in this study (or similar ones) noted any new or surprising burdens or benefits? What are these?

21. Have other people in this study (or similar ones) felt that, on balance, their participation was worthwhile? What seemed to make it feel worthwhile?

#### Understanding how the study is being carried out

22. How will I use this device in this study?

23. How often will I need to come in for the study?

24. Do people in the study find the experiments interesting and/or fun, or are they sometimes a bit boring?

25. Who will I interact with as part of the study and how often? How have other participants described their relationship with the research team?

26. How long has the trial been going on? How many people have been enrolled and how many are you planning to enroll? Are there any concerns about the study so far?

27. Who will have access to my medical records? How will my confidentiality be protected?

28. If I enter the study, will it require me to have extra tests, to attend more clinics and will it cost me extra money? (extra parking, extra medication?)

29. What happens if I am unable to come to or complete a study visit on a particular day (too tired, unable to find transportation, etc.)?

#### Understanding what happens after the study ends

30. At the end of the study, can I have the implant removed from my brain? Can I leave it in? What are the risks to either choice?

31. If the technology/device is successful, will I have access to it after the study is finished?

32. If the technology is successful for me, but the study is discontinued will I have access to it after the study is finished?

33. How will the results of the study be used?

Will I have access to the results of the study? If so, how and in what form?

#### Understanding possible conflicts of interest

34. Are you in charge of the study (the principal investigator)? If not, what’s your role in the study?

35. Who is funding the study? Who is providing the devices for the study?

36. Is there a payment by the technology company to the university/hospital or to you if I go on this study? Could you tell me how much money and is this usual? How is the money spent?

#### Understanding my right to join or not to join the study

37. Will I get treatment if I decide not to go into the study?

38. Do I have time to think about whether to join the study (a day or two, or a week)?

39. If I join the study, but later change my mind, how can I stop? Will I be penalized in any way?

40. Will participating in this study change my brain in ways that will prevent me or make me ineligible to participate in future studies or use future devices?

#### Concluding questions

41. Can I speak to someone who is already participating in this study or who has participated in a similar study in the past?

42. Are there other sources of information I can access? How can I learn more about the study? Who else could I speak with?

Your own questions: (Please write down any questions not listed).

## Data availability statement

The original contributions presented in this study are included in the article/supplementary material, further inquiries can be directed to the corresponding author.

## Ethics statement

The studies involving human participants were reviewed and approved by the University of Washington Human Subjects Division (HSD) who determined that the study qualifies for exempt status. The patients/participants provided their written informed consent to participate in this study.

## Author contributions

AS: conceptualization, original draft, review, and editing. SG and EK: conceptualization, review, editing, and supervision. NM, TB, ID, and EV: conceptualization, review, and editing. All authors approved the submitted version.
